# A Comprehensive Review on the Emerging Roles of Nanofillers and Plasticizers towards Sustainable Starch-Based Bioplastic Fabrication

**DOI:** 10.3390/polym14040664

**Published:** 2022-02-10

**Authors:** Shiou Xuan Tan, Andri Andriyana, Hwai Chyuan Ong, Steven Lim, Yean Ling Pang, Gek Cheng Ngoh

**Affiliations:** 1 Department of Mechanical Engineering, Faculty of Engineering, Universiti Malaya, Kuala Lumpur 50603, Malaysia; kva190014@siswa.um.edu.my (S.X.T.); andri.andriyana@um.edu.my (A.A.); 2 Center of Advanced Materials, Faculty of Engineering, Universiti Malaya, Kuala Lumpur 50603, Malaysia; 3 Future Technology Research Center, National Yunlin University of Science and Technology, 123 University Road, Section 3, Douliou, Yunlin 64002, Taiwan; onghc@yuntech.edu.tw; 4 Department of Chemical Engineering, Lee Kong Chian Faculty of Engineering and Science, Universiti Tunku Abdul Rahman, Kajang 43000, Malaysia; pangyl@utar.edu.my; 5 Centre of Photonics and Advanced Materials Research, Universiti Tunku Abdul Rahman, Kajang 43000, Malaysia; 6 Department of Chemical Engineering, Faculty of Engineering, Universiti Malaya, Kuala Lumpur 50603, Malaysia

**Keywords:** starch-based bioplastic, nanofiller, bioplastic fabrication, plasticizer, mechanism

## Abstract

Petroleum-based plastics are associated with environmental pollution problems owing to their non-biodegradable and toxic properties. In this context, renewable and biodegradable bioplastics possess great potential to replace petroleum-based plastics in mitigating these environmental issues. Fabrication of bioplastic films involves a delicate mixture of the film-forming agent, plasticizer and suitable solvent. The role of the plasticizer is to improve film flexibility, whereas the filler serves as a reinforcement medium. In recent years, much research attention has been shifted toward devising diverse methods for enhancing the performance of bioplastics, particularly in the utilization of environmentally benign nanoparticles to displace the conventional hazardous chemicals. Along this line, this paper presents the emergence of nanofillers and plasticizers utilized in bioplastic fabrication with a focus on starch-based bioplastics. This review paper not only highlights the influencing factors that affect the optical, mechanical and barrier properties of bioplastics, but also revolves around the proposed mechanism of starch-based bioplastic formation, which has rarely been reviewed in the current literature. To complete the review, prospects and challenges in bioplastic fabrication are also highlighted in order to align with the concept of the circular bioplastic economy and the United Nations’ Sustainable Development Goals.

## 1. Introduction

Fossil-fuel-derived plastics have vast applications ranging from food packaging, bottle drinks, furniture, household appliances and toys to medical equipment and even construction materials [1,2]. The high demand for plastics in these applications can be attributed to their low production cost and favourable properties such as high mechanical strength while maintaining a light weight, high resistance to degradation by water, chemicals, sunlight, and bacteria, as well as their capacity to provide adequate electrical and thermal insulation [3]. However, these conventional plastics are mainly derived from non-renewable petrochemical sources which can be degraded neither by the influence of solar radiation nor by the microbial decomposers [4]. As a consequence, plastic wastes continue to accumulate in the environment with the excessive usage of petroleum-based plastic products and hazardous chemical additives, which could cause severe impacts to the ecosystem.

Considering their non-biodegradability, for petroleum-based plastic wastes, recycling, landfill and incineration must be resorted to for their disposal [5]. It has been reported that only 7% of the 34 million tons of plastic waste produced per year was being recycled, while the remaining 93% ended up in landfills and oceans [2]. Plastic waste in the ocean could cause serious injuries, deformities or intoxication to marine animals when it is consumed due to the addition of endocrine disruptor compounds to the polymer matrix to enhance flexibility or colour properties [6]. The low recycling effort is mainly due to the high energy consumption of recycling. It was reported by Ross and Evans [7] that 18.9 MJ of energy was required to produce 1 kg of recycled material, which is tantamount to 23.5% of the cost required for manufacturing the same product from the virgin raw materials. On the other hand, the disposal of plastic waste in landfills has several disadvantages. Besides requiring a large land area, the major concerns are emission of toxic gases due to the absence of a gas collection system in normal practice and the probable leaching problem from landfill sites [8,9]. Furthermore, the incineration of plastic waste produces a net increase in carbon dioxide and may also emit hazardous gases such as sulfur dioxide, carbon monoxide and nitric oxide [10]. To solve and alleviate these economic and environmental problems brought about by petroleum-based plastics, considerable efforts have been directed towards the development of bioplastics which are biodegradable and more sustainable.

Bioplastics can be defined as biobased polymers derived from renewable resources or which are biodegradable and/or compostable naturally by microorganisms [11,12]. With growing concern about the economic and environmental problems caused by the utilization of petroleum-based plastics, the demand for bioplastics has increased tremendously in recent years. The global production capacity of bioplastic had increased by 38% per annum between 2003 and 2007, with a further projection that it would reach 3.45 million tonnes in 2020 [13]. The important characteristics of bioplastics include favourable mechanical and thermoforming properties, high gas and water vapor permeability, transparency and availability [14]. More importantly, the biodegradation products are usually non-toxic to the environment [1].

Apart from the generation of plastic waste, food waste generation was also alarming in Malaysia as it contributed up to 70% of the national municipal solid waste [15]. There is no comprehensive food waste management framework being established, which indicates that no specific method is available to dispose of food waste in a sustainable manner [16]. Despite the fact that 90% of food waste is biodegradable, reuse and recycle of the waste remains very low [15,17]. Recently, food wastes with high starch content such as mango seed [18], avocado seed [19], durian seed [20], jackfruit seed [12] and cassava peel [21] that can be utilized as bioplastic matrix has been extensively studied. These food wastes with high starch content have many advantages: they have a large supply availability and are low in price, biodegradable and renewable [22]. Nevertheless, native starch is brittle and has few mechanical properties, leading to poor film-forming capacity. In view of these drawbacks, most researches have focused on enhancing the functional properties and bonding strength of starch-based bioplastics through incorporation of less hazardous plasticizers and fillers to alleviate the brittleness of the materials [23].

As compared with petroleum-based plastics, bioplastics present three main disadvantages: the processing window, the performance and the cost. The narrow processing window, poor gas and water barrier properties, unbalanced mechanical properties, low softening temperature and weak resistivity of plastics have collectively limited their broader applications. Through substantial research work, the above limitations could be overcome by the introduction of nanofillers [24,25]. Meanwhile, the barrier properties of bioplastic could also be improved by incorporating suitable plasticizer. However, the cost of manufacturing bioplastics such as polylactic acid (PLA) and polyhydroxyalkanoate (PHA) is higher than that of conventional plastics. Therefore, blending them with biomaterial such as cellulose is a feasible way to minimize the cost [26].

The present article reviews the main classifications of bioplastics according to the feedstocks, the critical roles of emerging nanofillers and the selection of environmentally benign plasticizers in enhancing the properties of starch-based bioplastics. This is followed by an elucidation of the formation mechanism of starch-based bioplastics to provide a deeper insight into their properties down to the molecular level. The importance of the effects of different synthesizing parameters in the solvent-casting technique on the optical, mechanical and barrier properties of bioplastics is also discussed. Challenges and suggestions for future research are highlighted. These state-of-the-art reviews are still rarely available in the current literature. Therefore, this review could bridge the gap of the relevant scientific information required to transform the bioplastics industry into a more sustainable pathway in line with the circular economy.

## 2. Classifications of Bioplastics

**Figure 1:**
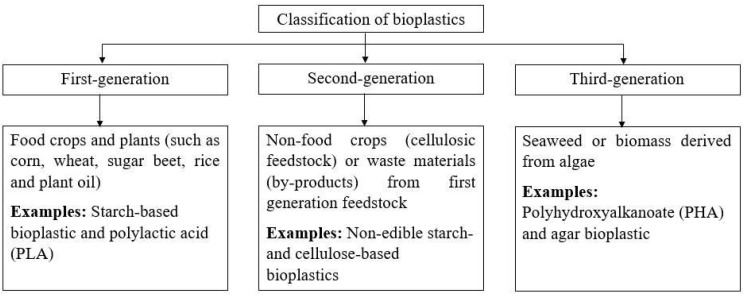
Classifications of bioplastics based on feedstock.

Currently, bioplastics have been developed in three different generations based on the types of feedstock used for the fabrication. The classification of bioplastics with relevant examples is depicted in Figure 1. Each generation of bioplastics has its respective advantages and disadvantages. Therefore, in-depth understanding of the properties of bioplastics is crucial to devise appropriate modification methods to suit different applications.

### 2.1. First-Generation Bioplastic

The feedstocks of the first-generation bioplastics are usually comprised of edible food crops or carbohydrate-rich plants such as corn or sugarcane. This is the most efficient feedstock since it requires the least land area to produce the highest yield based on highly matured plantation technology. Notable examples include corn, wheat, sugarcane, potato, sugar beet and rice. Starch-based bioplastics and PLA are examples of first-generation bioplastics derived from first-generation feedstocks.

**Figure 2:**
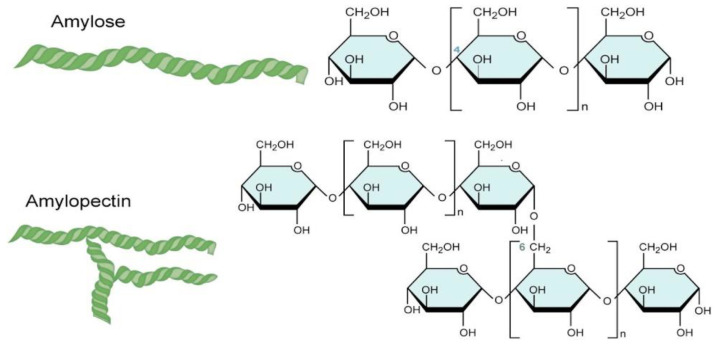
Structure of amylose and amylopectin in starch. Reprinted with permission from Ref. [28]. Copyright 2015 Elsevier Ltd.

Starch is a type of carbohydrate comprised of carbon, hydrogen and oxygen with a mole ratio of 6:10:5 [27]. It contains two main polysaccharide units: (i) linear chain amylose, which comprises 15−20% of starch; and (ii) branched amylopectin, which composes the majority of the remaining starch. Starch is also a natural semi-crystalline biopolymer of D-glucose [22]. The typical structures of amylose and amylopectin in starch are illustrated in Figure 2. Amylose units form the irregular amorphous region, while the well-ordered amylopectin region contributes to the crystallinity of starch. The concentration of amylose determines the gelling ability of starch, while the concentration of amylopectin controls the water-holding capacity of starch [28]. As the concentration of amylose increases, more starch will be converted into a gel structure and thus increase its elongation at break [27,28].

Native starch is challenging to use due to its brittleness, low mechanical properties and poor film forming capacity, as previously described. Plasticization and blending of starch with other polymers are often adopted to overcome these shortcomings [27]. In the fabrication of starch-based bioplastic, water is often used as a primary plasticizer because of its capability to hydrolyse the molecular bond structure of starch when heated together. The water content will also affect the oxygen permeability (OP) of starch-based bioplastic film. Under low-humidity conditions, the film can serve as an excellent barrier against oxygen transmission [27]. Besides, the hydrophilic nature due to the presence of numerous hydroxyl (O-H) groups and the poor long-term stability of native starch hinder its substitution for conventional plastic film. It has been reported that the crystalline amylopectin region obstructs the binding between plasticizers with the O-H groups, causing the starch matrix to retrograde after a certain period of time [29]. Therefore, fillers such as layered silicates, organic, inorganic and carbonaceous nanofillers can be incorporated into the matrix to reinforce the film, which is discussed in more detail in Section 3 [30].

In a bioplastic composite, starch acts as the matrix and plasticizer and filler as the plasticizing and reinforcement agents, respectively. The formation of starch-based bioplastics is related to starch gelatinization, whereby starch thickens in the presence of water after breaking down the intermolecular bonds of starch molecules. This allows the hydrogen bonding sites to engage with more water molecules. The starch granules dissolve irreversibly in water due to the plasticizer. Gelatinization simply turns a colloidal system from a temporary suspension to a permanent suspension, involving processes such as starch granule swelling, crystal or double helical melting and amylose leaching.

At the initial stage, water molecules enter the amorphous amylose region of starch granules, causing them to swell and expand. They are unable to enter the crystalline amylopectin region at ambient temperature. With an increase in the temperature, the additional heat energy melts the crystalline amylopectin region [31] and dissolves the amylose. Consequently, the number and size of crystalline regions decreases. At gelatinization temperature (75 °C), sufficient heat energy can break the hydrogen bond to allow more water molecules to enter and expand the starch granules. When amylose molecules leach into the surrounding water solvent, the granular structure disintegrates and forms a gel in the amylose matrix. A bioplastic composite process is initiated by mixing cracked starch granules with suitable plasticizer and filler [32]. The plasticizer molecules penetrate the starch granules and enlarge the cavities formed while destroying the inner hydrogen bonds of starch at high temperature with shearing. Starch–starch intermolecular bonding are destroyed and replaced with starch–plasticizer interactions [33]. As a result, the matrix becomes more elastic, which improves its elongation at break. On the other hand, the addition of suitable filler can fill the cavities in the matrix to form a denser bioplastic with strengthened mechanical properties [32].

One of the well-known bioplastics is PLA. PLA is a transparent plastic that possesses similar characteristics to common petrochemical-based plastics, such as polyethylene and polypropylene [34]. A wide variety of PLA structures, from semi-crystalline to totally amorphous, can be obtained depending on the feedstock from which the starch is derived. This bioplastic can be used for packaging applications as it is highly resistant to oil-based products and it can also act as flavour and odour barriers for foodstuffs [27,35]. Moreover, it fulfils the safety requirements for direct food contact with aqueous, acidic and fatty acids. In addition, cups, cutlery and food containers are commonly being manufactured using PLA [24]. However, high brittleness and poor oxygen barrier properties have restricted wider usage of PLA, making it less competitive when compared with conventional plastics, especially in the field of flexible films [24,36]. In order to enhance its properties, nanoclay has been added to promote the oxygen barrier of PLA [24], while various types of plasticizers such as poly(ethylene glycol) (PEG), oligomeric lactic acid (OLA) and tributyl citrate (TBC) can also be employed to reduce the brittleness of PLA [36].

**Figure 3:**
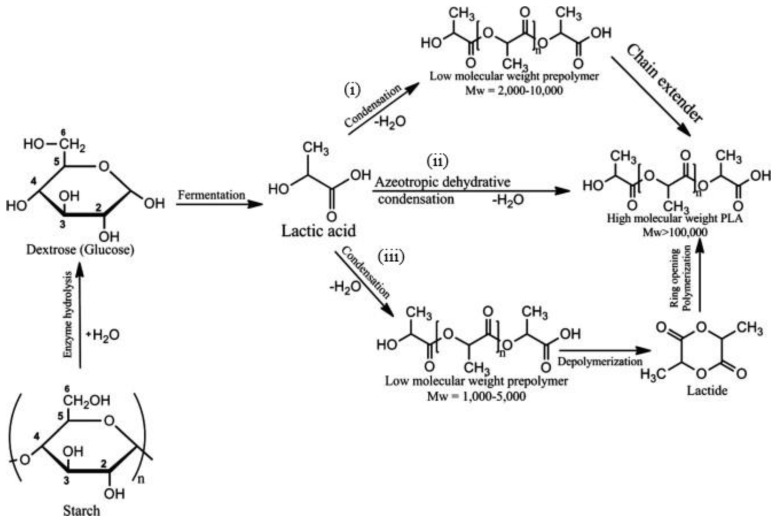
Fabrication routes of PLA. Reprinted with permission from Ref. [41]. Copyright 2015 Elsevier Ltd.

The fabrication of PLA constitutes a multi-step process. The process begins with the fermentation of hydrolysed sugar from starch to produce lactic acid monomers, and ends with the polymerization of lactic acid to produce PLA [27]. Lactic acid can be obtained from renewable carbohydrate sources such as corn, potato, whey and sugarcane through fermentation [37]. The three PLA fabrication routes shown in Figure 3 are (i) direct condensation polymerization; (ii) azeotropic dehydrative condensation; and (iii) ring-opening polymerization of lactide [38]. The direct condensation polymerization of lactic acid yields a brittle polymer with low molecular weight (2000 to 10,000 g/mol). Most of it has little use unless a chain extender is employed [34,38,39]. In the azeotropic dehydrative condensation of lactic acid, high-molecular-weight PLA (>100,000 g/mol) without the aid of chain extenders can be achieved. On the other hand, the ring opening polymerization of the lactide can engage appropriate catalyst to produce desirable PLA with designated molecular weight. As reported, the ratio and sequence of D- and L-lactic acid units in the final polymer chain could be controlled by the monomer and reaction conditions [38].

The first-generation feedstocks are generally rich in carbohydrate and are normally consumed by humans and animals. Continuous processing of the feedstock into bioplastic creates undesirable competition with food and animal feed supply [3,39,40]. Therefore, the second-generation bioplastic was developed to resolve this dilemma.

### 2.2. Second-Generation Bioplastic

In the fabrication of second-generation bioplastics, the feedstocks utilized are non-food crops such as lignocellulosic biomass. Lignocellulosic biomass derived from plants such as wood, straw, grass and bagasse is the most abundant biomass resource on earth [42]. One of the popular second-generation bioplastics is the cellulose-based bioplastic.

Cellulose is the most abundant natural resource on earth as it can be obtained from various living species such as plants (e.g., cotton, jute, flax, hemp, sisal, coir, ramie, abaca and kenaf), animals (e.g., tunicates), algae (e.g., red, green, grey and yellow-green), bacteria and even amoebas [43]. Cellulose consists of linear chains of D-glucose linked by β-(1,4)-glycosidic bonds and is organized into fibrils, which are surrounded by a matrix of lignin and hemicellulose [24,44]. Cellulose fibres are linked by a number of intra- and intermolecular hydrogen bonds. Since cellulose is insoluble in water and most organic solvents, it presents a challenge to bioplastic fabrication [43]. On the other hand, hemicelluloses located in secondary cell walls and composed of different sugar precursors (pentoses, hexoses and urgonic acids), are easier to hydrolyse as compared to cellulose. This is due to their branched structure (with short lateral chain), lower molecular weight and amorphous structure [44].

There are two major challenges in forming film materials directly from lignocellulosic biomass: its high immiscibility with water and the large amount of non-film forming fractions (lignin and hemicellulose) [42]. Several publications have reported the effectiveness of chemical modification to enhance the film-forming properties of cellulose and hemicellulose [45,46]. Chen and Shi [42] conducted a study in which sugarcane bagasse phthalate film was successfully synthesized via homogeneous esterification of sugarcane bagasse with phthalic anhydride as the inner plasticizer using 1-allyl-3-methylimidazium chloride as the reaction medium. The thermogravimetric analysis (TGA) results of the study revealed that the modified sugarcane bagasse had lower thermal stability than the original sugarcane bagasse. The contributing factor was the breaking down of the hydrogen bonds that held cellulose, hemicellulose and lignin molecular chains together when subjected to chemical modifications that facilitated the substitution of O-H groups by phthalic anhydrides.

### 2.3. Third-Generation Bioplastic

The most popular feedstocks used in third-generation bioplastic fabrication are algae and seaweed. The feedstocks can be cultivated naturally and they grow into enormous amounts in a short time span [40]. They do not directly compete with food resources and have a high growth rate and high growth tolerance in harsh conditions. These benefits render them a suitable alternative to replace first- and second-generation feedstocks [47,48]. Moreover, the bioplastic film derived from algae was found to be highly transparent, with high mechanical strength and flexibility [40]. Examples of third-generation bioplastics are PHA and agar bioplastic.

PHAs are polymers with desirable properties; they are mainly produced by microorganisms (cyanobacteria) and blue-green algae in the presence of excess carbon sources and a limited supply of essential nutrients such as nitrogen and oxygen [47,49]. PHA is chemically inert and hydrophobic and has a high melting point [49]. Different side chains of the alkanoate can be created by manipulating the growth conditions of the microorganism, particularly the composition of cultivation media, producing polymers with desirable properties. Examples of polymers that belong to the group of PHAs are 3-, 4-, 5-, and 6-hydroxyalkanoates, including polyhydroxybutyrate (PHB), polyhydroxyvalerate (PHV), or polyhydroxyhexanate (PHH) [50]. For the family of PHA, PHB and poly (3-hydroxybutyrate-co-hydroxyvalerate) (PHBV) are commercially available and have been produced at an industrial scale [24]. Pure PHB is very brittle and stiff due to its high crystallinity, and it has relatively high melting and glass transition temperatures [50]. As for PHBV, its excellent thermal resistance would have made it a potential replacement candidate for petroleum-based polyolefin. However, its high brittleness, low impact resistance and high production cost have unfavourably limited its application [24].

The other type of third-generation bioplastic is based on agar. Agar is a polysaccharide produced by several families of red seaweed (Rhodophyceae) such as *Gracilariaceae* and *Gelidiaceae*. It consists of two major components: agarose and agaropectin [51]. This type of hydrophilic colloid can form a reversible gel at low temperature [52]. Being the second largest genus of the red seaweed, *Gracilaria* comprised of more than 150 species distributed worldwide [53]. Because of its high gel strength, proteins, fatty acids and bioactive compounds, *Gracilaria changii* is in high demand for applications in the cosmetic, pharmaceutical and food industries [54].

Agar extracted from seaweed has great potential to be used as raw material for bioplastic film fabrication. As demonstrated in the study conducted by Hii et al. [48], agar extracted from red seaweed using the alkali (AEA) and photo-bleached (PBA) methods were added with sago starch and glycerol, respectively, to synthesize the bioplastic film. Both the AEA and PBA bioplastic films gave comparable yield, i.e., 10.68% vs. 9.83%. The employed alkali treatment would remove the sulfate groups such as 4-O-methyl-α-L-galactose and D-galactose-6-sulfate residues, leading to improved gelling ability. Moreover, the AEA method exhibited slightly better thermal stability based on the 74.84% vs. 80.52% weight loss from the TGA analysis (30 °C to 800 °C with nitrogen flow rate of 30 mL/min). Meanwhile PBA bioplastic film showed better tensile strength (3.07 MPa vs. 2.43 MPa) and percent elongation (3.27% vs. 2.48%) than the AEA bioplastic film. These findings confirmed the feasibility of synthesizing bioplastic films from agar.

## 3. Nanofillers for Property Enhancement in Bioplastics

Bioplastics reinforced with filler enhance the mechanical properties of starch but reduce its hydrophilicity [55]. In recent years, utilization of nano-sized fillers has bloomed in the fabrication of bioplastic due to their merits, such as low density, excellent mechanical properties, low abrasive nature and reactive surface for ease of modification [55,56]. Numerous studies had reported that nano-sized fillers have a larger surface area than the conventional micro-sized fillers, thus enhancing the properties due to better interfacial interactions with the polymer matrix [57]. Besides enhancing the mechanical and barrier properties, fillers are also capable of imparting specific functional properties to the bioplastics, e.g., electric conductivity or an antimicrobial character [23]. Another added advantage of filler with nanoscale is in retaining the inherent transparency of the film, especially for the neat matrix [55,58]. Several popular types of nanofillers utilized in starch-based bioplastics are layered silicates (nanoclay, nanosilica and montmorillonites (MMT)), organic nanofillers (cellulose), inorganic nanofillers (metal or metal oxide) and carbonaceous nanofillers (nanotubes, graphene, graphene oxide), which are listed in Table 1.

### 3.1. Layered Silicates

Nanoclays are also known as layered silicates [24]. They are readily available, environmentally friendly and of low cost. As a filler with high stiffness, nanoclay improves the mechanical properties of the soft polymer matrix by impeding the free movement of polymer chains. Nanoclay also behaves like a load-bearing constituent if the interfacial adhesion between the filler and the chains is sufficiently strong [73]. This was evidenced by the findings reported by Wahyuningtiyas and Suryanto [74] in which the tensile strength of bioplastic increased from 5.2 MPa to 6.3 MPa in the presence of 5 wt.% nanoclay. Apart from that, the research team discovered that nanoclay could also increase the microbial decomposition rate of bioplastic. For example, only half of the time is required by bioplastic reinforced with nanoclay to fully decompose, compared to a 12-day decomposition period required by the non-reinforced bioplastic. The higher decomposition rate was attributed to the aluminium ions in nanoclay, which acted as Lewis acids to catalyse the hydrolysis process [74]. Li et al. [61] also reported the improvement of the tensile strength of bioplastic from 48.96 MPa to 64.75 MPa after the addition of 5 wt.% of MMT. One point to note is that susceptibility to agglomeration is the unavoidable problem associated with the utilization of nanofiller in polymers. Therefore, ultrasound treatment was employed in the reported studies to disperse the nanofillers in the starch matrix. Homogeneous dispersion of nanofiller in the starch matrix could ensure even transfer of stress between the matrix and the nanofiller, thus enhancing the mechanical properties of polymer nanocomposites [61].

In order to utilize film for food packaging, oxygen and moisture transport must be minimized to extend food shelf-life [59]. In addition to improved mechanical properties, it can be observed from Table 1 that nanoclay showed barrier property (oxygen and moisture) improvement. Incorporation of nanoclay into the matrix would increase the path length and enhance the barrier properties of bioplastic to gases such as oxygen, carbon dioxide, organic vapours and moisture. The uniform dispersion of rigid impermeable nanoclay in the matrix might have hindered the diffusion of permeating molecules by forcing them to traverse and diffuse through a more tortuous path and thus reducing their mass transfer efficiency [24,73]. The antimicrobial effect of nanoclay against bacteria could be related to the release of ammonium salts, which affects bacteria-sensitive targets [75].

In the study by Liu et al. [60], retrogradation behaviour of TPS film was investigated with different surface properties of nano-SiO_2_. TPS film with hydrophilic nano-SiO_2_ had a lower retrogradation rate than that with hydrophobic nano-SiO_2_. During gelatinization of starch, the starch granules and crystalline structure were destroyed along with the breakdown of hydrogen bonds and the double helix structure. During short-term retrogradation, the amylose molecules started to crystallize and formed a crystal through a single helix structure. With longer retrogradation time, the amylopectin molecules gradually formed a crystal through a double helix structure. With the addition of nano-SiO_2_, the starch structure would be affected through interaction between O-H groups of starch and nano-SiO_2_. The addition of hydrophilic nano-SiO_2_ produced a V-type starch with a single helix structure. Formation of hydrogen bonds between O-H groups of hydrophilic nano-SiO_2_ and starch subsequently destructed the hydrogen bonds between the double helix structure of starch. In contrast, the double helix structure of starch remained with the addition of hydrophobic nano-SiO_2_, which was conducive to the regular arrangement of the double helix structure for crystallization. This indicated that the retrogradation degree of TPS was larger with hydrophobic nano-SiO_2_. Furthermore, the mobility of starch molecules was affected by the different surface properties of nano-SiO_2_. Hydrophilic nano-SiO_2_ has many O-H groups on its surface which could form hydrogen bonds with O-H groups of starch and reduce the mobility of starch molecules. On other hand, hydrophobic nano-SiO_2_ could not react with starch molecules as there is no O-H group on its surface. Therefore, TPS with hydrophobic nano-SiO_2_ had a higher retrogradation rate as a result of the faster movement of starch molecules during retrogradation.

### 3.2. Organic Nanofillers

Cellulose is one of the examples of organic nanofillers. There are three types of cellulose in nanoscale: cellulose nanocrystals (CNC), cellulose nanofiber (CNF) and bacterial nanocellulose [76]. CNC are needle-shaped cellulose particles with a typical diameter of 2–20 nm and a length varying between 100 nm to several micrometres. CNC particles are comprised of 100% cellulose and are highly crystallized, with 54% to 88% crystalline zones. The degree of crystallinity, dimensional diversity and morphology depend on the source of the cellulosic materials, the preparation conditions and the experimental techniques used [77]. The amorphous portion of the cellulose can be easily hydrolysed in strong acidic conditions to generate the individual crystallites. The particle size and properties of the isolated CNCs may vary depending on the cellulosic materials and reaction conditions during hydrolysis [55].

Similar to other fillers, CNCs can increase the stiffness of bioplastic based on their nanoscale dimension, low density, high surface area (≥100 m^2^/g), high aspect ratio of ≥100, high crystallinity and high inherent rigidity [56,62]. Xu et al. [63] revealed that CNC-reinforced starch film attained higher tensile strength than that without CNC reinforcement. In this case, a 10 vol.% CNC loading successfully improved the tensile strength of starch film by 129% as well as the barrier property of the starch film, with a 17% reduction in WVP. Another report on bioplastic fabrication by Chiulan et al. [78] revealed that 2 wt.% of bacterial cellulose derived from *G. xylinus* that was used as a reinforcement filler also had increased tensile strength and Young’s modulus up to 2 times and 2.1 times, respectively, as compared to that from non-reinforced bioplastic. Similar findings were also obtained in the study conducted by Noshirvani et al. [62]. Indeed, the mechanical properties of TPS-polyvinyl alcohol (PVA) composite film were improved with the addition of CNCs. Even at low loading of 3 wt.%, the uniform dispersion of CNC in the polymer matrix forced the gas molecules to traverse through a more tortuous path to pass through the bioplastic film and thus retarded the gas transmission and increased the mass transfer resistance of CNC-reinforced TPS-PVA composite film to water vapour. However, excessive CNC would cause particle agglomeration and reduce the effective content of CNC and thus facilitate the water vapour permeation.

Chitosan is another example of an organic nanofiller. It can be obtained from the deacetylation of chitin, which can be found in shell waste and skeletal materials of insects and crustaceans [64,79]. It is appealing to employ it as a filler as it serves the purpose of reusing the waste and being a biodegradable material. In addition, it is well-known for its antimicrobial properties. TPS/chitosan film was found to reduce the microbial growth of *S. aureus* and *E. coli.* The reduction of microbial activity was more pronounced at a higher chitosan concentration [64]. However, chitosan is insoluble in water, strong base solutions and some organic solvents such as alcohol and acetone. Therefore, an aqueous acidic solution such as hydrochloric acid (HCl) or acetic acid is required to dissolve the chitosan [80].

### 3.3. Inorganic Nanofillers

Metal or metal oxides are examples of inorganic nanofillers. Metallic nanoparticles such as Ag-NP are known to have antimicrobial and inhibitory activity against a variety of microorganisms such as bacteria, fungi or viruses [59,81,82,83]. From Table 1, Ag-NP was reported by Abreu et al. [59] to have a microbial effect against *S. aureus*, *E. coli* and *C. albicans.* Ag-NP would release Ag^+^ ions and penetrate into the bacteria, damaging the cell or disrupting the metabolic processes [84]. With the incorporation of hybrid nanofiller of Ag-NP/nanoclay into TPS, both the WVP and OP values were lower than the TPS with Ag-NP only. It was evidenced from the SEM images that incorporation of Ag-NP into nanoclay improved the clay dispersion in the starch matrix, which resulted in higher homogeneity of the film and thus better WVP and OP. Similar to Ag-NP, ZnO nanofillers also exhibit antimicrobial activity. In addition to their ability to be synthesized into different shapes [85], they are suitable to be incorporated into TPS films for food packaging application as they are considered Generally Regarded as Safe (GRAS) by the Food and Drug Administration (FDA) to be used in plastics and food contact materials [65]. From the study by Estevez-Areco et al. [65], TPS/ZnO could not inhibit the bacterial growth completely but it could delay the replication of bacterial growth, which could serve as a bacteriostatic agent.

### 3.4. Carbonaceous Nanofillers

CNT, GO and carbon black are examples of carbonaceous nanofillers. CNT is widely used as nanofiller in starch-based bioplastic owing to its superior mechanical (high tensile strength and Young’s modulus), electrical, magnetic, optical and thermal properties [61]. CNT can be produced by rolling sheets of carbon atoms (graphene) to form either single walled or multi-walled CNTs [33]. The study by Domene-López reported that as low as 0.5 wt.% amount of multi-walled CNTs could register an increment up to 327% in tensile strength, 2484% in Young’s modulus and also 82% of elongation at break. The augmentation of the mechanical properties was attributed to the good dispersion of multi-walled CNTs in the starch matrix. Furthermore, the findings also indicated that the better interfacial adhesion obtained could avoid the formation of holes and sustain a higher degree of deformation [86].

However, the natural insolubility of CNT in water causes self-association and a strong aggregation tendency, giving rise to low adhesion with the hydrophilic polymer matrix such as the starch matrix [87]. Functionalization of CNT through the adsorption of surfactant is the solution that could help in producing stable CNT dispersion. Alves et al. [68] had investigated the dispersibility of MWCNT by employing three different ionic surfactants, i.e., CTAB, sodium dodecyl sulphate (SDS), and sodium cholate (SC). TPS/MWCNT-SC showed the best dispersibility among them, producing a more homogeneous starch matrix, thus achieving the highest tensile strength and Young’s modulus. Nonetheless, the highest antioxidant activity and electrical conductivity were attained by the TPS with MWCNT-CTAB. SC interacted more tightly with the MWCNT surface than CTAB and SDS due to its rigid structure, so that the MWCNT surface was not so exposed. This subsequently reduced the availability of MWCNT to interact with the radical, which resulted in the lowest antioxidant activity [68]. In view of the electrical conductivity, TPS with MWCNT-SDS presented the lowest value, which might be due to agglomeration of nanotubes and weaker dispersibility, as observed in the SEM images. Higher adhesion of SC to the MWCNT surface gave rise to a blocking effect of charge transport in MWCNT network and diminished the contact points between nanotubes [88]. Therefore, the electrical conductivity value was lower. In addition, TPS with functionalized MWCNT could serve as an adsorbent for dye removal. From Table 1, TPS with MWCNT-AA, MWCNT-Fr and MWCNT-Valine was capable of removing dye. They are simple to prepare, cost little and are environmentally friendly. However, non-reusability is their chief drawback, as the dye adsorbed on the adsorbent surface cannot be separated completely from the absorbent for next use after dye removal.

## 4. Plasticizers Applied in Bioplastic Fabrication

Plasticizers consist of polar and low-molecular-weight molecules that are capable of disrupting intermolecular bonds between polymer chains and forming new bonds with O-H groups on the glucosidic units of polysaccharide chains. This facilitates chain movements and produces a more flexible material with a lower glass transition temperature. Thus, starch with added plasticizer can be easily processed to form thermoplastic derivatives. Plasticizers such as water, glycerol and sorbitol are commonly used in bioplastic fabrication [89]. However, it should be noted that the final properties of starch-based bioplastic are highly dependent on the ambient humidity. Hence, water is not recommended to be used directly as plasticizer. Otherwise, the high volatility of the water molecules would often produce a brittle film [30,33]. As for glycerol, it tends to migrate from starch films, leading to starch retrogradation after a prolonged storage period [89]. In a recent development, emerging plasticizers such as vegetable oil-based plasticizer, ionic liquid (IL) and deep eutectic solvent (DES) have attracted increasing attention. These novel plasticizers were reviewed, and their advantages and disadvantages are summarized in Table 2.

### 4.1. Vegetable Oil

Vegetable oil is composed primarily of fatty acid and glycerol. It is highly biodegradable and renewable. Many edible vegetable oils such as palm oil, soybean oil and rapeseed oil have been utilized as plasticizers. This has raised a tremendous concern as the use of edible oil, as plasticizer feedstock tends to incur food supply competition leading to a food security threat. Moreover, environmental issues such as deforestation will likely occur if a massive propagation of plants producing edible oil is to take place [36]. To resolve these potential shortcomings, inedible oils have been proposed as alternatives.

**Figure 4:**
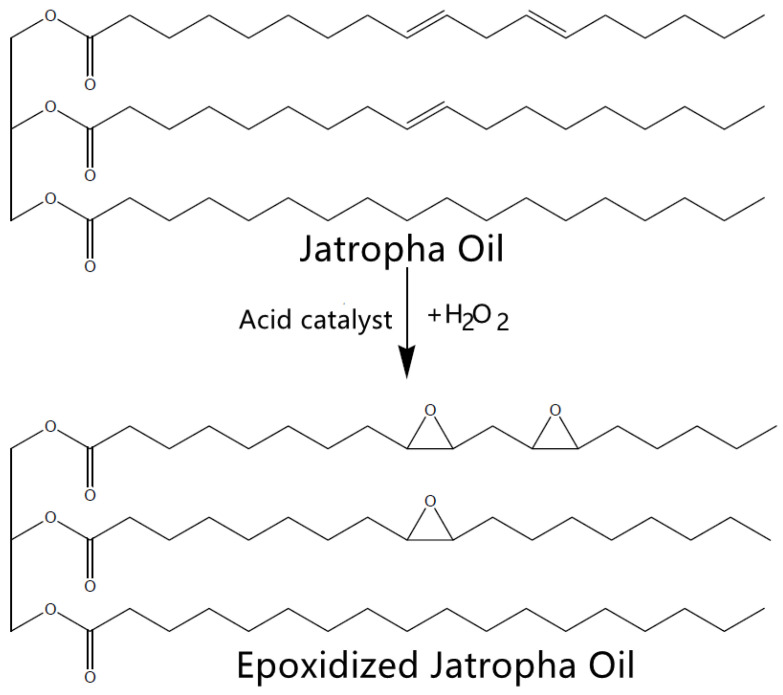
Epoxidation of *Jatropha* oil. Reprinted with permission from Ref. [36]. Copyright 2017 MDPI.

Epoxidized vegetable oils are often used as plasticizers instead of pure vegetable oils. The epoxidized vegetable oils have greater molecular weight and a bulkier structure than the pure vegetable oils which have helped to resist migration by minimizing volatility in the polymeric matrix [95]. Epoxidation is an oxidation reaction of adding oxygen atoms to carbon–carbon double bonds to form carbon–oxygen bonds, usually together with an acidic catalyst and H_2_O_2_. Unsaturated fatty acids are the primary target during the epoxidation of vegetable oils due to the higher amount of carbon–carbon double bonds [36]. For example, *Jatropha* oil, which consists of high unsaturated fatty acid, including oleic (C18:1) and linoleic (C18:2) acids, is suitable to be converted into epoxidized *Jatropha* oil before being used as plasticizers in bioplastics fabrication. A typical epoxidation reaction of *Jatropha* oil is presented in Figure 4.

Vegetable oil can also be used as an anti-microbial agent. In the algae biofilm synthesized by Othman et al. [40], cinnamon bark oil was utilized as a co-primer and anti-microbial agent to improve the life-span of the biofilm. The oil contained about 50% cinnamaldehyde, an organic compound that occurs naturally as a predominant trans (E) isomer, providing cinnamon with a unique flavour and odour [96]. From the soil burial test, the algae biofilm incorporated with cinnamon bark oil showed less biodegradation potential compared to the normal algae biofilm. The addition of 5% cinnamon bark oil to the biofilm demonstrated a slower biodegradation rate and reduced pore percentage. This has proven the improvement of the insect repellent and the anti-microbial properties of cinnamon in biofilm protection [97].

### 4.2. Ionic Liquid (IL)

Ionic liquids (ILs) are organic salts of bulky organic cation and a smaller organic or inorganic anion, with a melting point below the boiling point of water at 100 °C [91,98]. Due to their non-volatility (negligible vapour pressure), non-flammable properties, good ionic conductivity, and high thermal, chemical and electrochemical stability, ionic liquids are a superior replacement for organic solvents [90,91]. For instance, the cellulose that could be dissolved in 1-butyl-3-methylimidazolium chloride (BMIMCl) was incorporated with a plasticizer to prepare the cellulose film [98]. The interaction of cellulose and BMIMCl involved oxygen and hydrogen atoms from the hydroxyl (O-H) groups of cellulose [99]. The oxygen and hydrogen atoms from cellulose could serve as electron donors and electron acceptors, respectively. In a corresponding fashion, the BMIM^+^ acted as electron acceptor centres while Cl^−^ as electron donor centred. Upon interaction, the intermolecular hydrogen bonds were cleaved, resulting in the dissolution of cellulose with BMIMCl [100,101]. It was reported that the quality of the cellulose/BMIMCl composite films depended on the concentration of the ionic liquid retained during water removal from the ionic gel, which in turn played a critical role in determining the mechanical properties of the films [98]. The optimum mechanical properties were attained at 40% BMIMCl concentration. Apart from the hydrogen bonding interactions, the stacking interactions between intra- and inter-sheets of the cellulose layers might have also oriented the cellulose chains [98]. When the cellulose chains were being staked uniformly, the stacking interactions between the cellulose chains would be enhanced, resulting in an increase in tensile strength and elongation at break [100,102]. It has been reported that when the BMIMCl concentration was increased beyond the optimum level at 40%, the advantages of BMIMCl would diminish to a typical plasticizer. At a higher concentration of more than 40%, BMIMCI would enable the molecules to penetrate the cellulose chains and obscure the interactions between them. This would create the sliding effect in tensile property resulting in decreased tensile strength but increased elongation at break. On the other hand, it was insufficient for the BMIMCl concentration at less than 40% to effectively plasticize the cellulose chains due to the poor distribution of stress and very low elongation at break.

Even though ILs have always been considered as green solvents, uncertainty about their environmental impacts had also been raised [98]. For instance, there was a controversy over the sustainability of pyridinium- or imidazolium-based ILs as they could become more hazardous than other organic solvents. From the ecotoxicological test, pyridinium- and imidazolium-based ILs were found to show moderate ecotoxicity to bacteria, algae, and invertebrates. They would pose harmful impacts to the aquatic ecosystem if released into the open environment [103]. Moreover, ILs are difficult to mass-produce as doing so would incur high production costs due to the time-consuming fabrication and energy-intensive purification steps involved [90]. The effort in searching for alternative green solvents leads to the discovery of deep eutectic solvent (DES), which demonstrates similar physical and chemical properties as IL.

### 4.3. Deep Eutectic Solvent (DES)

Deep eutectic solvents are mixtures of two or more compounds which consist of hydrogen bond acceptors (e.g., halides salts of quaternary ammonium or phosphonium cations) and hydrogen bond donors (e.g., citric acid, urea or glycerol). The bond acceptors and the bond donors react with each other through strong hydrogen bond interactions to produce DES with a lower melting point and vapour pressure but higher thermal stability than their individual components [90,104]. The charge delocalization, occurring through hydrogen bond formation between the components, is responsible for the lowering of the melting point of the DES compared to its individual components [104].

DES has been employed to replace the traditional imidiazolium-based ILs. Although exhibiting similar performances as conventional IL, DES is comparatively cheaper, safer and easier to prepare in large amounts via a simple mixing process [93,94]. Because of its renewable and non-toxic properties, DES has been utilized as plasticizer and crosslinking agent in bioplastic fabrication [93,105]. In particular, choline-chloride-based DESs are favourable for starch modification as they can interact strongly with OH groups from glycosidic units, decrease chain interactions and plasticize the polymer [91]. The employment of a DES plasticizer comprised of choline citrate/glycerol in the fabrication of TPS film was reported by Zdanowicz et al. [89]. They discovered that the tensile strength of the film increased while elongation at break decreased as the choline citrate content in DES was increased. The work reported an inversely proportional relationship between choline citrate content in the TPS film and the sorption degree of the film. In addition, it also indicated a direct correlation whereby the higher amount of citrate anion in the system can lead to a higher degree of crosslinking. The work further confirmed the occurrence of a partial crosslinking reaction between the polysaccharide and DES components via Fourier transform infrared spectroscopy (FTIR) spectra and dynamic mechanical analysis. Among the highlights of the findings reported was the non-retrogradation of the TPS/choline citrate/glycerol film even after 12 months of storage. The covalent-bonded compound could also exert resistance to migration or evaporation from the polymeric matrix, which substantiated the superiority of reactive plasticizer such as DES over the conventional plasticizer (e.g., glycerol). Therefore, it is still inherently safe to apply DESs synthesized bioplastic film in the food packaging or agricultural industry [89].

## 5. Critical Factors Affecting Properties of Bioplastic Using Solvent-Casting Technique

The important optical properties of bioplastic film usually refer to its colour and opacity. The opacity of the film indicates the amount of visible wavelength that can pass through the film [106]. Besides that, the mechanical property of the synthesized film materials is also essential in various applications to prevent films from cracking [42]. Mechanical properties of bioplastic film include the tensile strength, Young’s modulus and elongation at break. High tensile strength is generally desirable, and the value can be fine-tuned depending on the proposed application of the film [55]. A lower Young’s modulus value is preferred as it imparts higher elasticity to the bioplastic sample [22]. Elongation at break is usually used as an indication of the bioplastic flexibility [18]. Other barrier properties affecting the bioplastic film include WVP, OP and moisture uptake. Several main factors that influence the optical, mechanical and barrier properties of the bioplastic are plasticizer loading, filler loading, processing temperature of bioplastic solution, concentration of chitosan solvent as well as concentration and composition of starch.

### 5.1. Plasticizer Loading

Bioplastic often encounters fragility issues due to its high intermolecular forces, which render it very rigid. With the addition of plasticizer, the moisture content of the starch solution is enhanced, and thus the starch granule can move more freely. The elasticity and flexibility of bioplastic is enhanced through the weakening of the internal hydrogen bonds among polymer chains and increasing the molecular spacing [18,107]. Therefore, plasticizer plays an important role in improving the flexibility, softening and elongation properties of the bioplastic [20]. This had been witnessed in the fabrication of bioplastic film by Lubis et al. [12], whereby an increment in the concentrations of plasticizer sorbitol increased the adsorption intensity of the O-H group on the bioplastics. As the sorbitol molecules slipped between amylose–amylopectin–chitosan chains, the interaction between the polymers weakened and led to a reduction in the tensile strength [108]. A similar trend was observed by Cifriadi et al. [13] on the enhancement in tensile strength of starch-cellulose bioplastic film when the glycerol as plasticizer reached an optimum loading of 37.5%. On the other hand, inadequate plasticizer loading could lead to imperfect plasticizing efficiency causing the starch molecules to become more brittle [21].

Increment of the plasticizer loading during the fabrication of bioplastic increases the elongation at break of bioplastic. This relationship was proven based on the bioplastic synthesized by Ginting et al. [108]. The elongation at break of the bioplastic was found to increase with increasing sorbitol concentrations. They attributed this observation to the low molecular weight of sorbitol when compared to polymeric compounds such as starch and chitosan. The addition of the plasticizer would increase the free movement space for the polymer molecules. The optimum conditions for bioplastic fabrication with various plasticizers are compiled in Table 3. Glycerol and sorbitol are two of the most commonly utilized plasticizers due to their ability in reducing the internal hydrogen bonding of the polymer molecules and thus increasing their intermolecular distance [20]. The range of plasticizer loading to achieve the optimum mechanical properties was reported to be between 1.5 wt.% to 45 wt.%. It can be observed from Table 3 that by using different fillers on the same feedstock and plasticizer, the amount of plasticizer required would also be different. In the bioplastic fabrication using cassava starch as matrix and glycerol as the plasticizer, a higher concentration of glycerol was required for ZnO as compared to that using nanoclay. This was explained by the requirement of higher plasticizer loading to compensate for the lower filler loading. It has been reported by the researchers that the tensile strength of the film was greater by using ZnO instead of nanoclay due to the formations of stronger alkane, C-O, C=C and C-C bonds with the addition of ZnO. In another case involving chitosan as the filler and sorbitol as the plasticizer, durian seed starch bioplastic required higher optimum sorbitol loading compared to sago starch bioplastic (i.e., 45 wt.% vs. 25 wt.%). However, the sago starch bioplastic achieved higher tensile strength than the durian seed starch bioplastic (46.71 MPa vs. 10.63 MPa). The presence of fat content in the durian seed was believed to form complexes with amylose and to cause the granule surface to be enveloped by hydrophobic fat. Amylose was then inhibited from being released from the granules during gelatinization and the gelling ability of starch was reduced [20,28].

### 5.2. Filler Loading

The addition of suitable fillers can affect the optical properties of bioplastic. When Almeida et al. [104] prepared chitosan film using DES as plasticizer and curcumin as the additive, they discovered that an increment in the curcumin concentration caused an increment in the colour parameters (total colour difference (Δ*E*), chromaticity parameters, a* (red-green) and b* (yellow-blue)) and a decrement in the lightness (L*). Similar trends were noted in other chitosan films incorporating different additives such as tea polyphenols [110] or cinnamon essential oil [111]. Moreover, the opacity of the film was found to increase with an increase in the curcumin loading. Embedding curcumin pigment into the interspaces of chitosan film obstructed the light transmission. However, Jacquot et al. [112] believed that the colour change in the chitosan film was initiated by the microwave heating process whereby neoformed compounds that appeared in the Maillard reaction were responsible for the film colouration. The Maillard reaction is a very complex reaction between carbonyls and amines which occurs spontaneously during food processing and storage [113].

Unlike plasticizer, the addition of filler could improve tensile strength and Young’s modulus while reducing elongation at break. Filler promotes the formation of strong hydrogen bonds between O-H groups on the interface of both the filler and starch matrix. The formation of the stronger molecular bonding increased the tensile strength and Young’s modulus of bioplastic. Conversely, elongation at break of bioplastic decreased as intermolecular bonding distance was reduced [18]. Strong filler–matrix compatibility and good filler dispersion also accounts for better reinforcement and thus provides more promising mechanical properties. On the other hand, aggregation of filler should be prevented since it might create an heterogeneous phase, feature discontinuities and decrement in the desired mechanical properties of the film [55].

Modification of property in bioplastic was reported in the work of Agustin et al. [55] using CNCs as filler to reinforce the bioplastic film in various loadings (0 to 15 wt.%). The CNC loading increased the tensile strength and Young’s modulus, whereas the elongation at break was decreased from 33.1% (without CNC) to 4.2% (at 10 wt.% maximum loading of CNC). Expectedly, the increment of the CNC loading increased the stiffness of the film, which then decreased its elasticity [55]. Strong hydrogen bonds between O-H groups from starch and O-H and COOH groups from cellulose in CNC contributed to higher strength and lowered the elongation at break [21]. A similar observation was also obtained by Ginting et al. [19] in their bioplastic film fabrication utilizing chitosan as filler. All the findings reported thus far unanimously agreed that mechanical properties were affected primarily by the number and types of the chemical bonding on the polymer matrix such as covalent, hydrogen and Van der Waals bonds. This was clearly demonstrated by the enhancement of hydrogen bonds and the compactness of intermolecular bonds in the bioplastic with the addition of chitosan to reduce the intermolecular bond distance. This fabrication pathway successfully produced a stronger and more rigid bioplastic [1,19]. Other studies also reported the same findings, which include the fabrication of bioplastic film reinforced with microcrystalline cellulose (MCC) by Maulida and Tarigan [21] and the study undertaken by Wahyuningtiyas and Suryanto [10] using nanoclay. The tensile strength of bioplastic decreased when nanoclay was added beyond its optimum loading of 5 wt.%.

It is worth noting that the density of bioplastic has a large impact on its mechanical properties. A denser bioplastic usually possesses higher tensile strength, which improves its mechanical properties. It is essential to ascertain the density of bioplastic so that the most appropriate packaging and efficiency can be selected [10]. In the study conducted by Wahyuningtiyas and Suryanto [10], the density of bioplastic increased with the addition of nanoclay (filler) as the spatial arrangement of molecules in the composite became more compact [109]. The density of bioplastic is also affected by the degree of crystallinity of the polymer. Maulida and Tarigan [21] obtained a reduction in the density of bioplastic as MCC was added. This anomaly trend was due to the introduction of ultrasonic treatment, which resulted in an increase in the amorphous region in the bioplastic fabrication [114]. A higher amorphous fraction was attributed to a lower density of bioplastic due to non-uniformity and less dense molecules. Lower density bioplastic exhibited more open structures and was more susceptible to penetration by fluids such as H_2_O, O_2_ or CO_2_ [21].

Minimal moisture uptake of film is favourable, which indicates that the film is water-resistant [55]. Starch-based bioplastics usually have poor resistance to moisture since starch is a hydrophilic polymer [115]. Therefore, the addition of appropriate filler can also improve the water resistance of starch-based bioplastics [55]. Hydrophilic behaviour of starch-based bioplastic can be retarded by mixing starch with hydrophobic biopolymers such as cellulose and chitosan [12]. In the fabrication of bioplastic film conducted by Agustin et al. [55], moisture uptake of the film decreased from 13.8% to 10% with the addition of CNC loading from 0 to 10 wt.%. The decrement in the moisture uptake could be attributed to the formation of hydrogen bonding between O-H groups on the interface of both CNCs and the starch matrix. The resulting hydrogen-bonded network of cellulose with the starch in the composite could prevent the formation of voids, which allows water molecules to pass through [55]. A similar trend was found by Maulida and Tarigan [21], who observed that water uptake decreased as the MCC loading was increased. Furthermore, the cellulose possessed strong hydrogen bonding, which rendered it difficult to bond with water. Another similar observation was discovered by Kartika et al. [18] which showed that increasing the clay content would decrease the water uptake of bioplastics.

The thermal stability of bioplastic film, which can be characterized using TGA, is an indicator of its durability at high temperature applications [55]. A decline in the thermal stability of the bioplastic film was reported when the maximum weight-loss rate temperature (T_max_) decreased from 323.75 °C to 277.26 °C as the CNC loading increased from 0 wt.% to 15 wt.% [55]. This was caused by the inherent low thermal stability of CNCs after being subjected to sulfuric acid hydrolysis. The significant reduction in crystallinity of CNC after sulfuric acid hydrolysis also reduced the thermal stability of bioplastics [116].

The bioplastic synthesized by Anggraini et al. [109] was also more vulnerable to higher processing temperatures with increased chitosan loading. The higher processing temperature can affect the appearance and mechanical properties of bioplastic with low thermal stability. As reported, bioplastic became more fragile after being heated at 100 °C for 10 min. When heated at a higher temperature of 125 °C for the same duration, the bioplastic started to become more brittle and could be easily broken. Moreover, the colour of bioplastic would turn darker after being heated at the higher processing temperature, which indicated a reduction in mechanical properties. The deterioration of the thermal stability of bioplastic could be explained by the interaction between chitosan and starch in the bioplastics. If the interaction between starch chains is weak and the starch molecules are not in the optimal position, a higher processing temperature can disintegrate or inflict damage on the bioplastics [64].

In a study on TPS film conducted by Salaberria et al. [117], the water vapour transmission rate (WVTR) decreased to around 85 g∙mm∙m^−2^∙day^−1^ from 114 g∙mm∙m^−2^∙day^−1^ with the addition of 5 wt.% chitin nanocrystals in the TPS film. Interestingly, with the same amount of 10 wt.% chitin nanofibers, the WVTR value was on par with the pure TPS film. A further increase of the nanofibers to 20 wt.% would surpass the WVTR value of the pure TPS film. The findings recommended low loading of chitin nanocrystal and nanofiber contents as they could create a more tortuous path to block water molecules from passing through. Otherwise, the presence of excessive contents of chitin nanocrystal and nanofiber would increase the WVTR. With regards to the OP of pure TPS film, its value decreased by roughly 30% and 25% with the addition of 10 wt.% chitin nanocrystal and nanofiber, respectively. Incorporating filler into TPS film has the advantage of imparting the antimicrobial properties against fungi and bacteria. This was proven by the growth inhibition of the *A. niger* exerted by chitin nanocrystal- and nanofiber-reinforced TPS film. At filler loading at 20 wt.%, chitin nanofiber-reinforced TPS film exhibited better inhibition effect compared to the chitin nanocrystal, i.e., fungal growth inhibition (FGI, %), 96% vs. 89%. Unlike the antimicrobial property, the inhibition rate was not affected by the concentration of chitin nano-materials. When chitin nanocrystals of 10 wt.% and 20 wt.% were incorporated into TPS film, there was only a slight FGI difference of 2% (87% vs. 89%, respectively). Furthermore, the mechanism demonstrated by the antimicrobial property of chitin was not conclusive and is still under intense research [117].

### 5.3. Concentration of Chitosan Solvent

Chitosan is commonly incorporated into starch-based bioplastics as a co-polymer or filler to reduce the water sensitivity as well as enhance the mechanical and barrier properties of film [118]. In addition, it is considered as GRAS by the FDA, making it an ideal candidate for food packaging material [79]. Since only chitosan can be dissolved in aqueous acidic solution with a pH of less than 6.3, an aqueous acetic acid solution is commonly used as the chitosan solvent [79]. HCl can also serve the same function as acetic acid. Ginting et al. [20] studied the effect of the concentration of chitosan solvent on the mechanical properties of bioplastic. The chitosan solvent used various HCl concentrations ranging from 0.9 vol.% to 1.3 vol.%. The highest and lowest tensile strengths of 10.63 MPa and 2.65 MPa, respectively, were detected at the two extremes of HCl concentrations. Higher HCl concentration would cause lower solubility of chitosan. Due to the limited solubility of chitosan in inorganic acids, it could dissolve in 1% HCl but not at all in sulfuric acid and acid phosphate. At 0.9 vol.% HCl concentrations, the solvent provided a better distribution of chitosan in the polymer matrix. The chitosan added into the starch solution mixture was able to fill up the voids in the bioplastic to form a dense bioplastic with increased resilience and tensile strength. The interaction between chitosan and starch suspension supports was observed through FTIR based on the increasing number of O-H and N-H groups on bioplastic, which was attributed to the interaction of the amylose–amylopectin–chitosan. The presence of these hydrogen bonds would enhance the tensile strength as well as Young’s modulus of the bioplastic. Similar to the trend shown by the tensile strength, the highest Young’s modulus of 129.51 MPa and its lowest value at 29.07 MPa were attained at 0.9 vol.% and 1.3 vol.% HCl concentrations, respectively. Since Young’s modulus was inversely proportional to elongation at break, the highest elongation at break was obtained at 1.3 vol.% HCl concentration [20].

### 5.4. Processing Temperature of Bioplastic Solution

The processing temperature at 90 °C achieved the optimum mechanical properties for bioplastic film as determined by Ginting et al. [19]. Higher processing temperature improved the tensile strength of the film as the intermolecular bond on starch chains weakened and triggered the breaking down of the amylose long chain bond. Moreover, higher processing temperatures promoted homogeneity in the bioplastic which rendered the structure more compact, thus improving the tensile strength. Nevertheless, higher processing temperatures can also have adverse effects on the plasticizer volatilization from the bioplastic. It caused bioplastic to become more arid and easier to be torn. This, in turn, reduced the elasticity of bioplastic and increased the Young’s modulus of the bioplastic [19].

It was also discovered that increasing the processing temperature of the bioplastic solution beyond the optimum range deteriorates its tensile strength due to the weakening of the intermolecular bonds in starch chains. Excessive heating would break the glycosidic bonds (bonds between monomers) in amylose [108]. This was agreed by Haryanti et al. [119], in whose study increased processing temperature was found to promote the depolymerization of the amylose chains and subsequently decrease the amylose content. Amylose plays an important role in the gel formation and the production of compact thin layer (film). A reduction in amylose content would lower the cohesiveness of bioplastics formation and thus decrease its tensile strength [108].

For elongation at break, it increases with the increasing processing temperature of the bioplastic solution. As previously discussed, the addition of sorbitol increased the elongation at break of bioplastic [108]. The increase in the kinetic energy of the molecules was attributed to the increase in processing temperature. As the vibrations of the molecules increased, more free volumes are created to allow larger molecular chain rotation [120].

### 5.5. Concentration and Composition of Starch

As mentioned previously in Section 2.1, starch consists of two main polysaccharide units, namely amylose and amylopectin. Amylose content was reported to have major impacts on the mechanical [121] and optical properties [122] of the synthesized bioplastic film. Sifuentes-Nieves et al. [121] reported that high amylose contents could modify the mechanical properties of glycerol-plasticized starch films to a greater extent, giving higher tensile strength and higher Young’s modulus but lower elongation at break. These findings were substantiated through the higher gelatinization and glass transition temperatures of the amylose fraction of starch [123]. However, tensile strength and Young’s modulus would decrease while the elongation at break increase with increasing plasticizer concentrations above 15 wt.% regardless of the type of starch employed.

In addition, the amount of lipids in the starch interferes with the opacity of the film. An increase in starch concentration in the film increases the lipid concentration. Meanwhile, the lipid particles could scatter the visible light through the film, causing it to be more opaque [106]. Potato starch film is more transparent than wheat and corn starch films as the latter contains a higher amount of lipids [122]. The opacity and the colour properties of the bioplastic film are dependent on the starch original properties (amylose/amylopectin ratio, size and shape of starch granules) and its concentration. A higher starch concentration contained more amylose in its matrices, and thus the film appeared to be more opaque [122]. The researchers further investigated the effect of starch with different amylose and amylopectin ratios on the optical properties of the synthesized bioplastic film involving corn, wheat and potato starches. Starch with lower amylose content or higher amylopectin content favours the formation of greater homogeneity, as well as denser and thinner film. Among the three types of starch studied, corn starch film was opalescent and thickest at 112 µm, while potato starch film was the least heterogeneous, most transparent and thinnest at 55 µm. Generally, the thinner film is favourable as it can ease the retraction of starch gel during drying. As a summary, the film thickness is dependent on the amylose/amylopectin ratio, which in turn determines the opacity and transparency of film.

The biodegradability of bioplastic was evaluated by Hasan and Rahmayani [1], who incubated the bioplastic film in the culture medium of *Pseudomonas aeruginosa*. The film which contained higher starch content had a colour change in the media after 10 days of incubation and was completely biodegraded after 30 days. Owing to the presence of glucosidic bonds in the amylose and amylopectin units, bioplastic film with greater starch content could be biodegraded easily through the hydrolysis mechanism.

## 6. Challenges and Potential for Future Sustainable Development

Based on the review presented, nanofiller has a demonstrated ability to enhance the mechanical, optical and barrier properties of bioplastics. However, the scientific literature reporting on the utilization of nanofillers in starch-based bioplastics is still limited. Considering the knowledge gaps waiting to be bridged by the fundamental studies in dealing with the reaction mechanism of nanofiller on starch-based bioplastics, more in-depth and related studies should be carried out. As for the preparation of nanofiller, the process remains complex and not economically feasible. These have restricted the potential of reinforced starch-based bioplastics in replacing conventional petroleum-based plastics [30]. As discussed in Section 5.2, chitin has intrinsic antimicrobial properties against fungi and bacteria, but the associated mechanism leading to the properties has yet to be confirmed [117]. This opens up an opportunity for further exploration to gain an insight into how filler could act as antimicrobial agent besides its reinforcement role. Until now, it is still difficult to obtain insightful information pertaining to economic feasibility on energy consumption and cost analysis for bioplastic fabrication. Hence, it is essential to take into account the thermo-economic aspects of pretreatment and feedstock preparation, including the usage of environmentally benign chemicals in bioplastic fabrication as part of environmental mitigation strategies.

Uniform dispersion of materials is desired when nanofiller is incorporated in bioplastic as its effect can be manifested in its physical and mechanical properties. Nano-sized particles tend to agglomerate with each other, and this problem can be resolved with the aid of ultrasound in dispersing nanomaterials in the polymer [10]. However, scientific data elucidating the effect of ultrasound (for instance, sonication and ultrasonic amplitude) on the dispersion of nanomaterials is rare and not well established. This could prompt more research efforts towards the investigation of ultrasound on mechanical, optical, barrier and biodegradability properties of bioplastic. In addition, the life-cycle assessment analysis of bioplastic from production to its ultimate disposal or recycling is also worth studying to ascertain its sustainability in the context of the circular economy [124,125].

The leaching of plasticizer during storage or end-user applications is also a major concern which could restrict the utilization of plasticizers in essential sectors such as the medical, pharmaceutical and food packaging industries. Plasticizer has comparatively low molecular weight (300–600 g/mol), which could potentially migrate from packaging materials into packaged food, thereby becoming indirect “food additives” [126]. Ideal plasticizers can be derived from renewable sources with desirable properties such as high biodegradability, chemical stability, absence of toxins, and no or little leaching or migration during usage or aging. Nevertheless, the leaching of the plasticizer during storage and its toxicity is still under scrutiny. In view of this, the migration possibility or mechanism in plasticized bioplastics should be investigated so that appropriate remediation or pollutant removal strategies can be devised [36].

When using starch-based bioplastics as food packaging materials, their hydrophilic nature exhibits a low water vapour barrier, which is responsible for poor processing ability, high brittleness, vulnerability to degradation, limited long-term stability and poor mechanical properties [11]. In addition, the potential of nanoparticles to migrate from packaging materials to packaged food should also be taken into consideration [73]. Therefore, risk assessment such as a toxicology study should be conducted. Storage tests should also be performed for the safe usage of packaging films based on bioplastics before they can be launched into the mass market to replace conventional packaging materials.

## 7. Conclusions

The biodegradable and green starch-based bioplastic is highly desirable to replace the conventional petroleum-based plastic due to its sustainability and environmental friendliness together with its readily available starch sources from food waste. The optical properties of starch-based bioplastics are affected by the amylose and amylopectin contents in starch. Lower amylose or higher amylopectin content resulted in more transparent film. On the other hand, more lipid content in starch can cause the film to become more opaque. The addition of environmentally benign nanofillers in bioplastic fabrication can overcome two main inadequacies of starch-based bioplastics, namely, high water affinity and poor mechanical properties. Filler-incorporated bioplastic has an enhanced tensile strength and Young’s modulus due to the large surface area and aspect ratio, which increase the hydrogen bonding between filler–amylose–amylopectin molecular chains. Moreover, barrier properties can be improved by forcing the permeable molecules to follow a more tortuous path which will impede their diffusion. The addition of plasticizer weakens the structure of bioplastic and decreases the tensile strength while increasing the elongation at break of bioplastic. From the collated findings, it can be deduced that tensile strength is closely related to the added fillers, while elongation at break is associated with the addition of plasticizer. Overall, utilizing agricultural or food waste for the production of starch-based bioplastic can maximize their values and reduce the accumulation of hazardous petroleum-based plastic wastes. Commercialization of starch-based bioplastic is highly plausible if it can strike a balance among its mechanical properties, production cost, toxicity and an acceptable level of biodegradability. Therefore, a synergistic relationship between the addition of fillers and plasticizers is crucial to establish in future research studies.

## Figures and Tables

**Table 1:** List of nanofillers incorporated in starch-based films with the reported findings or properties enhancement.

Nanofiller	Proposed Application	Findings/Enhancement as Compared to the Control Film	Ref.
Layered silicates			
Nanoclay	Food packaging film	Reduction of water vapour permeability (WVP) by 14% Reduction of OP by 15% Presence of microbial growth against *C. albicans* Reduction of microbial growth against *S. aureus* and *E. coli* (bacteriostatic effect)	[59]
Nanoclay	Packaging material	Improvement of tensile strength from 5.2 to 6.3 MPa Increase in moisture absorption from 44.44% to 69.58% Complete degradation of thermoplastic starch (TPS)/nanoclay film on the 6th day	[10]
Nanosilica (nano-SiO_2_)	Packaging material	TPS film with hydrophilic nano-SiO_2_ had lower retrogradation rate than that with hydrophobic nano-SiO_2_.	[60]
MMT	Packaging material	Improvement of tensile strength by 32% with MMT loading of 5 wt.% Improvement of Young’s modulus from 2338 to 3237 MPa Improvement of surface hydrophobicity of film (from 51.97° to 67.77°) Reduction of moisture uptake by 11%	[61]
Organic nanofillers			
Cellulose nanofibers (CNF)	Packaging material	Improvement of tensile strength by 33% with CNF loading of 3 wt.% Improvement of Young’s modulus from 2338 to 3173 MPa Improvement of surface hydrophobicity of film (from 51.97° to 53.89°) Reduction of moisture uptake by 13%	[61]
Cellulose nanocrystals (CNC)	Packaging film	Reduction of water absorption and water solubility by 21% and 50% with CNC loading of 20 wt.%, respectively Reduction of WVP by 8% with CNC loading of 15 wt.%.; WVP value increased with 20 wt.% CNC loading Optimum tensile strength of 4.59 MPa at 10 wt.% CNC loading; reduction in tensile strength with addition of 15 and 20 wt.% CNC loadings	[62]
Cellulose nanocrystals (CNC)	Food packaging film	Improvement of tensile strength by 56% with CNC loading of 10 vol.% Reduction of WVP by 17%	[63]
Chitosan	Packaging film	Improvement of tensile strength by 17% with chitosan loading of 10 wt.% Improvement of Young’s modulus by 13% Reduction of WVP by 35% TPS/chitosan film had higher opacity than TPS film Reduction of microbial growth against *S. aureus* and *Escherichia coli*	[64]
Chitosan	Packaging film	Optimum tensile strength of ~6.79 MPa at TPS/chitosan ratio of 4:6 Higher biodegradation rate with increase of starch content	[1]
Inorganic nanofillers			
Zinc oxide (ZnO) nanorods	Food packaging film	Improvement of tensile strength (47 to 90 MPa) and Young’s modulus (2.1 to 3.2 MPa) Slight reduction of elongation at break from 50% to 47%. Reduction of WVP by 42%. Improvement of antimicrobial activity against *E. coli* from 1.5 × 10^7^ to 9 × 10^5^ CFU/mL	[65]
Silver nanoparticles (Ag-NP)	Active packaging film	Improvement of tensile strength (2.8 to 9.0 MPa) and Young’s modulus (50 to 530 MPa) Reduction of EB from 63% to 20% Improvement of antibacterial activity against *E. coli* from 5.0 × 10^7^ to 1.5 × 10^6^ CFU/mL Film with AgNP disintegrated slower than the control film in soil (after 2 weeks vs. after 1 week)	[66]
Ag-NP	Food packaging film	Reduction of WVP by 16% Reduction of OP by 11% No microorganism growth against *S. aureus*, *E. coli* and *C. albicans* (microbiostatic effect)	[59]
Ag-NP/nanoclay	Food packaging film	Reduction of WVP by 33% Reduction of OP by 35% No microorganism growth against *S. aureus*, *E. coli* and *C. albicans* (microbiostatic effect)	[59]
Carbonaceous fillers			
Multi-walled carbon nanotubes (MWCNT)	For packaging and electroconductive applications	Improvement of tensile strength by 327% and Young’s modulus by 2484% at MWCNT loading of 0.5 wt.% Highest electrical conductivity of 56.3 S/m with 5 wt.% loading as compared to control film (1.08 × 10^−3^ S/m) Shifting of thermal degradation temperature to lower temperature with increasing MWCNT loading	[67]
Multi-walled carbon nanotubes functionalized with cetyltrimethylammonium bromide (MWCNT-CTAB)	Production of conductive film	Improvement of 2,2′-azino-bis-(3-ethylbenzothiazoline-6-sulfonic acid) (ABTS) radical scavenging activity (from ~2.5% to 30.2% after 1.5 h) Improvement of electrical conductivity (from 2.03 × 10^−6^ S/m to 14.75 S/m)	[68]
Multi-walled carbon nanotubes functionalized with ascorbic acid (MWCNT-AA)	As adsorbent for removal of methylene blue (MB) dye from aqueous solution	Enhancement of thermal stability Suitable to be used as adsorbent for removal of MB dye but not reusable	[69]
Multi-walled carbon nanotubes functionalized with ascorbic acid (MWCNT-AA)	As adsorbent for removal of methylene range (MO) dye from aqueous solution	Enhancement of thermal stability Suitable to be used as adsorbent for removal of MO dye but not reusable	[70]
Multi-walled carbon nanotubes functionalized with fructose (MWCNT-Fr)	As adsorbent for dye removal from aqueous solution	Film was too brittle for tensile test	[33]
Multi-walled carbon nanotubes functionalized with Valine (MWCNT-Valine)	As adsorbent for removal of copper ions from aqueous solution	Enhancement of thermal stability Suitable to be used as adsorbent for removal of copper ions but not reusable	[71]
Graphene oxide (GO)	Food packaging film	Improvement of tensile strength (from 57.97 to 76.09 MPa) and Young’s modulus (from 20.59 to 35.91 MPa). Slight reduction of EB from 6.60% to 3.13%. Enhancement of thermal stability Improvement of surface hydrophobicity of film (from 71.33° to 112.04°) Improvement of water vapour permeability Starch/gelatin/GO film had lower biodegradability than the control film (~30% vs. 50%) after 6 weeks of soil burial degradation.	[72]

**Table 2:** Advantages and disadvantages of plasticizers.

Plasticizer	Examples	Advantages	Disadvantages	Ref.
Vegetable oil	*Jatropha* oil Castor oil	Biodegradable Renewable	Edible vegetable oil competes with food supply	[1,36]
IL	1-allyl-3-methylimidazolium chloride 1-butyl-3-methylimidazolium chloride	Non-volatile due to negligible vapour pressure Non-flammable Good ionic conductivity High thermal stability High chemical stability High electrochemical stability	Difficult to prepare High production cost (time consuming fabrication and purification)	[90,91,92]
DES	Deep eutectic salts based on choline chloride	Cheaper to produce Easy to prepare in large quantity Less toxic than IL	Sometimes biodegradable	[91,93,94]

**Table 3:** Optimum reaction conditions for fabrication of starch-based bioplastics using solvent-casting technique.

Sources of Starch	Filler; Starch to Filler Ratio	Filler; Optimum Loading	Plasticizer; Optimum Loading	Processing Temperature (°C)	Tensile Strength (MPa)	Young’s Modulus (MPa)	Elongation at Break (%)	Moisture Uptake (%)	Ref.
Corn	-	CNC; 10 wt.%	Glycerol; 3 wt.%	70	26.80	898	4.20	10	[55]
Avocado seed	Chitosan; 7:3	-	Glycerol; 0.2 mL/g	90	5.10	36.36	14.03	-	[19]
Cassava peel	-	MCC Avicel PH101; 6 wt./v%	Sorbitol; 20 wt.%	70	9.12	-	-	70 *	[21]
Cassava	-	Nanoclay; 5 wt.%	Glycerol; 1.5 vol.%	80	13.50	47	-	-	[10]
Cassava	-	ZnO; 0.6 wt.%	Glycerol; 25 wt.%	85 ± 5	22.30	-	220 *	-	[57]
Jackfruit seed	Chitosan; 8:2	-	Sorbitol; 25 wt.%	88.82	13.52	-	-	-	[12]
Sago	-	Chitosan; 20 wt.%	Sorbitol; 25 wt.%	70	46.71	-	0.32	130.31	[109]
Durian seed	-	Chitosan; 15 wt.%	Sorbitol; 45 wt.%	70	10.63	129.51	8.21	-	[20]
Yellow pumpkin	Chitosan; 6:4	-	Castor oil; 15 wt.%	-	6.79	6.09	13.45	-	[1]
Mango seed	-	Clay; 6 wt.%	Glycerol; 25 wt./v%	-	5.66	-	43.43	32.28	[18]

* denotes for values estimated from charts presented in the original reference.

## Data Availability

The data presented in this study are available on request from the corresponding author.
